# Evaluation of carotid plaque echogenicity based on the integral of the cumulative probability distribution using gray-scale ultrasound images

**DOI:** 10.1371/journal.pone.0185261

**Published:** 2017-10-04

**Authors:** Xiaowei Huang, Yanling Zhang, Long Meng, Derek Abbott, Ming Qian, Kelvin K. L. Wong, Rongqing Zheng, Hairong Zheng, Lili Niu

**Affiliations:** 1 Paul C. Lauterbur Research Center for Biomedical Imaging, Institute of Biomedical and Health Engineering, Shenzhen Institutes of Advanced Technology, Chinese Academy of Sciences, Shenzhen, China; 2 Shenzhen College of Advanced Technology, University of Chinese Academy of Sciences, Shenzhen, China; 3 Department of Ultrasound, Third Affiliated Hospital, Sun Yat-sen University, Guangzhou, China; 4 Centre for Biomedical Engineering, and School of Electrical and Electronic Engineering, University of Adelaide, Adelaide, Australia; 5 School of Medicine, Western Sydney University, Campbelltown, New South Wales, NSW, Australia; Kurume University School of Medicine, JAPAN

## Abstract

**Objective:**

Carotid plaque echogenicity is associated with the risk of cardiovascular events. Gray-scale median (GSM) of the ultrasound image of carotid plaques has been widely used as an objective method for evaluation of plaque echogenicity in patients with atherosclerosis. We proposed a computer-aided method to evaluate plaque echogenicity and compared its efficiency with GSM.

**Methods:**

One hundred and twenty-five carotid plaques (43 echo-rich, 35 intermediate, 47 echolucent) were collected from 72 patients in this study. The cumulative probability distribution curves were obtained based on statistics of the pixels in the gray-level images of plaques. The area under the cumulative probability distribution curve (AUCPDC) was calculated as its integral value to evaluate plaque echogenicity.

**Results:**

The classification accuracy for three types of plaques is 78.4% (kappa value, *κ* = 0.673), when the AUCPDC is used for classifier training, whereas GSM is 64.8% (*κ* = 0.460). The receiver operating characteristic curves were produced to test the effectiveness of AUCPDC and GSM for the identification of echolucent plaques. The area under the curve (AUC) was 0.817 when AUCPDC was used for training the classifier, which is higher than that achieved using GSM (AUC = 0.746). Compared with GSM, the AUCPDC showed a borderline association with coronary heart disease (Spearman *r* = 0.234, *p* = 0.050).

**Conclusions:**

Our experimental results suggest that AUCPDC analysis is a promising method for evaluation of plaque echogenicity and predicting cardiovascular events in patients with plaques.

## Introduction

Cardiovascular diseases significantly threaten human health and are the primary cause of death and disability worldwide [1]. Most myocardial infarctions, strokes, and acute coronary syndromes are caused by the rupture of unstable atherosclerotic plaques [2]. In recent years, growing evidence has been presented to support the association between plaque echogenicity and its vulnerability [3,4]. Echolucent plaques are dominated with lipid content, less calcification, less fibrous tissue, and tend to be more prone to rupture [5,6]. In addition, previous studies have demonstrated that plaque echolucency is associated with coronary events and future stroke [7,8]. Therefore, it is of significant interest to evaluate the plaque echogenicity, which may contribute to the identification of unstable plaques and for predicting cardiovascular events.

It is well-known that ultrasound imaging is a non-invasive technique for carotid atherosclerosis plaque examination. Ultrasound measurement of plaque echogenicity can provide a risk factor for predicting cardiovascular events [9–11]. Visual classification has been used to assess the plaque echogenicity in many previous studies [11–13], however, the results are operator-dependent. Recent studies have shown that computer assisted methods of plaque characterization using B-mode images can provide measurements in predicting clinical outcome [4,14–19]. Percentage white (PW) has been proposed as a metric for evaluation of echogenicity in carotid plaques, but it needs an intensity threshold to determine which pixels are echogenic (white) [14]. The process of PW feature extraction is relatively complex because the intensity threshold of each image is different. Texture analysis has been utilized to characterize carotid atherosclerotic plaques in symptomatic and asymptomatic patients [15–17], and it shows promise in the assessment of plaque echogenicity by combining the morphological characteristics of plaques [20]. However, the extraction of texture features requires high computational complexity. Recent studies have indicated that computerized measurement of the gray-scale median (GSM) is an objective and useful metric for assessment of the plaque echogenicity [4,18,19]. It is worthwhile noting that GSM is the fiftieth percentile of the probability distribution of gray-scale pixels, and it ignores the details of the probability distribution of plaques. Shankar et al. proposed a method to model the statistics of the pixels in the gray-level images of soft and hard plaques [21]. The cumulative probability distribution curves showed significant trends for these two types of plaques. Therefore, we suggest that the area under the cumulative probability distribution curve (AUCPDC) may be an effective parameter for evaluating plaque echogenicity.

The aim of this study is to examine whether the AUCPDC analysis is a useful method for the evaluation of plaque echogenicity and to further compare its efficiency with GSM.

## Subjects and methods

### A. Patients

The study protocol was approved by the Institutional Review Board of the third affiliated hospital of Sun Yat-sen University (Guangzhou, China). All participants provided written informed consent.

From September 2013 to March 2016, a total of 130 carotid plaques were collected from 74 volunteers, and 5 controversial plaques were excluded after visual classification by two sonographers. The remaining 125 carotid plaques (43 echo-rich, 35 intermediate and 47 echolucent plaques) from 72 volunteers were used in the in the following analysis.

### B. Clinical and biochemical analyses

Blood samples were collected after an overnight fast for analysis of total cholesterol, triglyceride, high density lipoprotein cholesterol, low density lipoprotein cholesterol, apolipoprotein A1, apolipoprotein B100, fasting plasma glucose, and HbA_1c_. The diagnostic criteria for hypertension was defined as systolic blood pressure ≥ 130 mmHg and/or diastolic blood pressure ≥ 80 mmHg or current use of antihypertensive agents. Diabetes was defined as fasting plasma glucose level of ≥ 7.0 mmol/L, and/or 2-hour plasma glucose value of ≥ 11.1 mmol/L, and/or HbA_1c_ level of ≥ 6.5%, and/or treatment with either hypoglycemic agents or insulin [22,23].

### C. Images acquisition and preprocessing

Ultrasound images of carotid plaques were collected by a sonographer that has 5 years of experience in vascular imaging using an Aplio XG (SSA-790A) (Toshiba Medical Systems, Japan) equipped with a 5–12 MHz linear-array transducer (PLT-805AT). The carotid artery was examined with the head tilted slightly upward in the mid-line position. The transducer was manipulated so that the near and far walls were parallel to the transducer footprint, and the lumen diameter was maximized in the longitudinal plane.

According to the criteria of the European carotid plaque study group, plaques were classified into three different types: echolucent, intermediate and echo-rich plaques [12]. The visual classification of plaque echogenicity was independently performed by two sonographers with at least 5 years of experience in vascular imaging, and a kappa value (*κ*) was calculated to evaluate the between-observer agreement.

### D. Image normalization

The ultrasound system settings (e.g. system gain, time gain compensation etc.) can impact the brightness and contrast of the B-mode images. In this study, all images were normalized according to the scheme proposed by Sabetai et al [24]. After normalization, the GSM of the blood range from 0 to 5, whereas the GSM of adventitia range from 185 to 195.

### E. Statistics of the pixels in gray-level images of plaques

In this study, the plaque was manually segmented by one operator in the gray-level image, and the statistics of the pixels of plaques were obtained. Then, the AUCPDC analysis and GSM analysis were performed for each plaque based on their pixel statistics.

### F. Gray-scale median

Here, we let *x* represents the gray scale pixel value, *f*(*x*) is the probability density function of *x*, and *f*(*x*) can be calculated as follows:

$$f{\left(x\right)}_{}^{}{=}_{}^{}\frac{\mathrm{The}\;\mathrm{number}\;\mathrm{of}\;\mathrm{pixels}\;(\mathrm{gray}\;\mathrm{value}\;=x)}{\mathrm{Total}\;\mathrm{number}\;\mathrm{of}\;\mathrm{pixels}\;(\mathrm{gray}\;\mathrm{value}\;\mathrm{range}\;\mathrm{from}\;0\;\mathrm{to}\;255)}.$$ (1)

**Fig 1:**
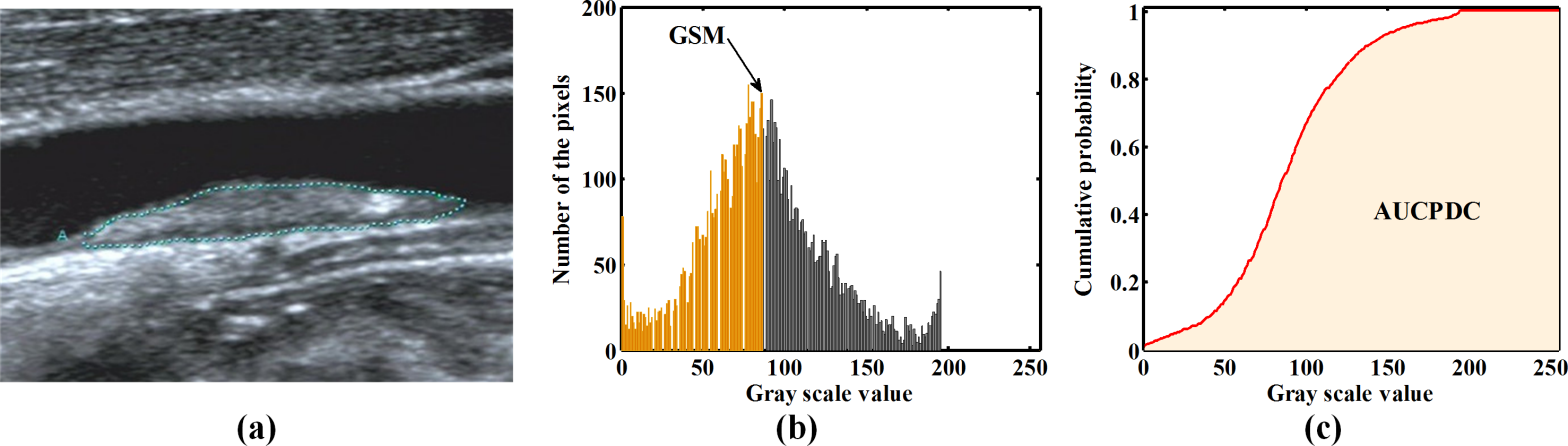
The GSM and AUCPDC of representative image of plaque. (a) Representative image of plaque; (b) When the orange area is 0.5, the horizontal coordinate value is GSM of the plaque illustrated in (a); (c) the shaded areas are the AUCPDC of the plaque illustrated in (a). Note that GSM = gray-scale median; AUCPDC = area under cumulative probability distribution curve.

The GSM is defined as follows:

$$0.5_{}^{}{=}_{}^{}\sum_{x=0}^{\text{GSM}}f(x)$$ (2)

where *f*(*x*) is defined in Eq (1). A direct expression of GSM is shown in Fig 1B.

### G. Area under cumulative probability distribution curve

For each plaque, the cumulative distribution function *F*(*x*) of the gray scale distribution, can be expressed as follows,

$$F(x_{i})=\sum_{x=0}^{x_{i}}f(x)\;x=0,1,2,\ldots ,254,255.$$ (3)

The AUCPDC is measured as follows,

$${\text{AUCPDC}}_{}^{}{=}_{}^{}\sum_{x=0}^{255}F(x).$$ (4)

Fig 1C illustrates the AUCPDC (shaded area).

### H. Classification

The k-nearest-neighbor (KNN) classification was performed for classifying the three different types of plaques based on AUCPDC or GSM. In order to improve the reliability of classification, a leave-one-out cross validation was implemented in this study. The kappa statistic (κ) was calculated to evaluate the agreement between visual classification and the KNN classification. Furthermore, the receiver operating characteristic (ROC) curves for the KNN classifier were developed to compare the ability of AUCPDC and GSM in identifying echolucent plaques.

### I. Intra-operator agreement

In order to examine the intra-operator agreement, the AUCPDC analyses were performed at two different times within a 2-month period. Based on the same visual classification, a total of 45 plaques (15 echolucent, 15 intermediate and 15 echo-rich plaques) were randomly selected from the same original images, and the manual plaque segmentation was carried out again by the same operator. The intra-operator agreement was evaluated according to the method proposed by Bland and Altman [25].

### J. Bootstrapping for estimating Youden's index *J*

Youden's index *J* [26] is a single statistic that can summarize the performance of a diagnostic test, and it is defined as:

$$J_{}^{}{=}_{}^{}{\text{sensitivity}}_{}^{}{+}_{}^{}{\text{specificity}}_{}^{}\;\text{-}\;1.$$ (5)

It has been widely utilized in many studies to evaluate the accuracy of diagnostic tests and the performance of risk assessment model [27,28].

Bootstrap is a useful tool to provide statistical inference to estimate the accuracy and the precision of any statistic through resampling with replacement from the original datasets[29,30]. In this study, the bootstrapping is implemented to estimate the 95% confidence intervals (CIs) of Youden's index *J*.

### K. Statistical analyses

All statistical analysis was performed with PASW Statistics 18 and all values were presented as the mean value ± SD, or real number of patients with the percentage in parentheses. Spearman's rank correlation analysis was also implemented between the GSM, AUCPDC and the status of hypertension, diabetes, coronary heart disease (CHD). Youden's index *J* was calculated using MedCalc statistical software.

## Results

### A. Patient characteristics

The baseline characteristics of the study population are shown in Table 1. Among a total of 72 patients (51 male, 70.1%; age, 69.7 ± 8.7 years), 46 (63.9%) patients had hypertension, 31 (43.1%) had diabetes and 27 (37.5%) had CHD.

**Table 1:** Baseline characteristics of 72 patients.

Characteristics	Total (n = 72)
**Age, mean (SD)**	69.7 ± 8.7
**Male gender, n (%)**	51 (70.1)
**Hypertension, n (%)**	46 (63.9)
**Smoking, n (%)**	29 (40.3)
**Diabetes, n (%)**	31 (43.1)
**CHD, n (%)**	27 (37.5)
**SBP (mm Hg)**	136.5 ± 22.9
**DBP (mm Hg)**	77.0 ± 12.5
**BMI (kg/m^2^)**	22.2 ± 2.7
**TC (mmol/L)**	4.26 ± 1.12
**TG (mmol/L)**	1.43 ± 1.14
**HDL-C (mmol/L)**	1.04 ± 0.27
**LDL-C (mmol/L)**	2.66 ± 0.95
**ApoA1 (g/L)**	1.19 ± 0.29
**ApoB100 (g/L)**	1.06 ± 0.38
**FBG (mmol/L)**	6.65 ± 2.87
**HbA_1c_ (%)**	6.31 ± 1.84

CHD = coronary heart disease; SBP = systolic blood pressure; DBP = diastolic blood pressure; BMI = body mass index; TC = total cholesterol; TG = triglycerides; HDL-C = high-density lipoprotein cholesterol; LDL-C = low-density lipoprotein cholesterol; ApoA1 = Apolipoprotein A1; ApoB100 = Apolipoprotein B100; FBG = fasting blood glucose.

### B. Visual classification

The classification of the carotid plaques (*n* = 130) into three different types showed a good agreement between two experienced sonographers (Table 2). The between-observer reproducibility was 96.15% (*κ* = 0.942). A total of 5 controversial plaques were excluded, and the 125 consensual plaques were retained in the following analysis.

**Table 2:** Visual classification of 130 carotid plaques by two experienced sonographers.

		Sonographer 2			
		Echoluent	Intermediate	Echo-rich	Total
**Sonographer 1**	**Echoluent**	47	0	0	47
	**Intermediate**	3	35	0	38
	**Echo-rich**	1	1	43	45
	**Total**	51	36	43	130

*κ* = 0.942.

### C. The area under cumulative probability distribution curve of plaque

**Fig 2:**
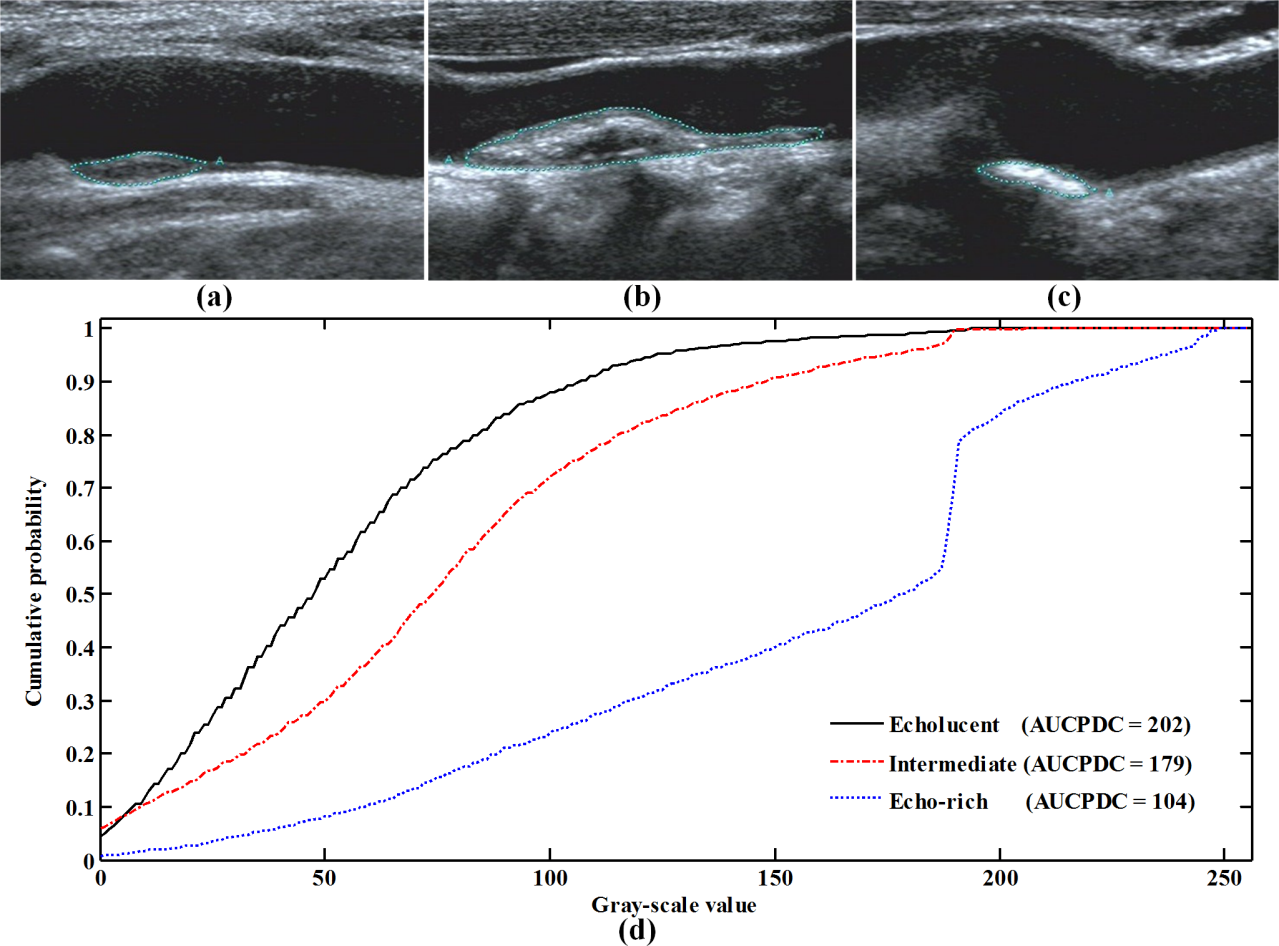
The AUCPDC of representative echoluent, intermediate and echo-rich plaques. The echoluent, intermediate and echo-rich plaques in (d) were defined in the original images (a), (b) and (c), respectively. Note that AUCPDC = area under cumulative probability distribution curve.

Fig 2 shows the AUCPDC analysis of representative echoluent, intermediate and echo-rich plaques. The AUCPDC of echolucent plaques were largest, followed by intermediate plaques and echo-rich plaques (Fig 2D). The AUCPDC showed a statistical significance among the echo-rich, intermediate, echolucent plaques (130 ± 22 vs. 177 ± 18 vs. 207 ± 21, *p* < 0.001). When type 1 denoted echo-rich, type 2 intermediate type 3 echolucent, the mean AUCPDC had a correlation with the types of plaques (Spearman *r* = -0.856, *p* < 0.001).

### D. Carotid plaque classification

**Fig 3:**
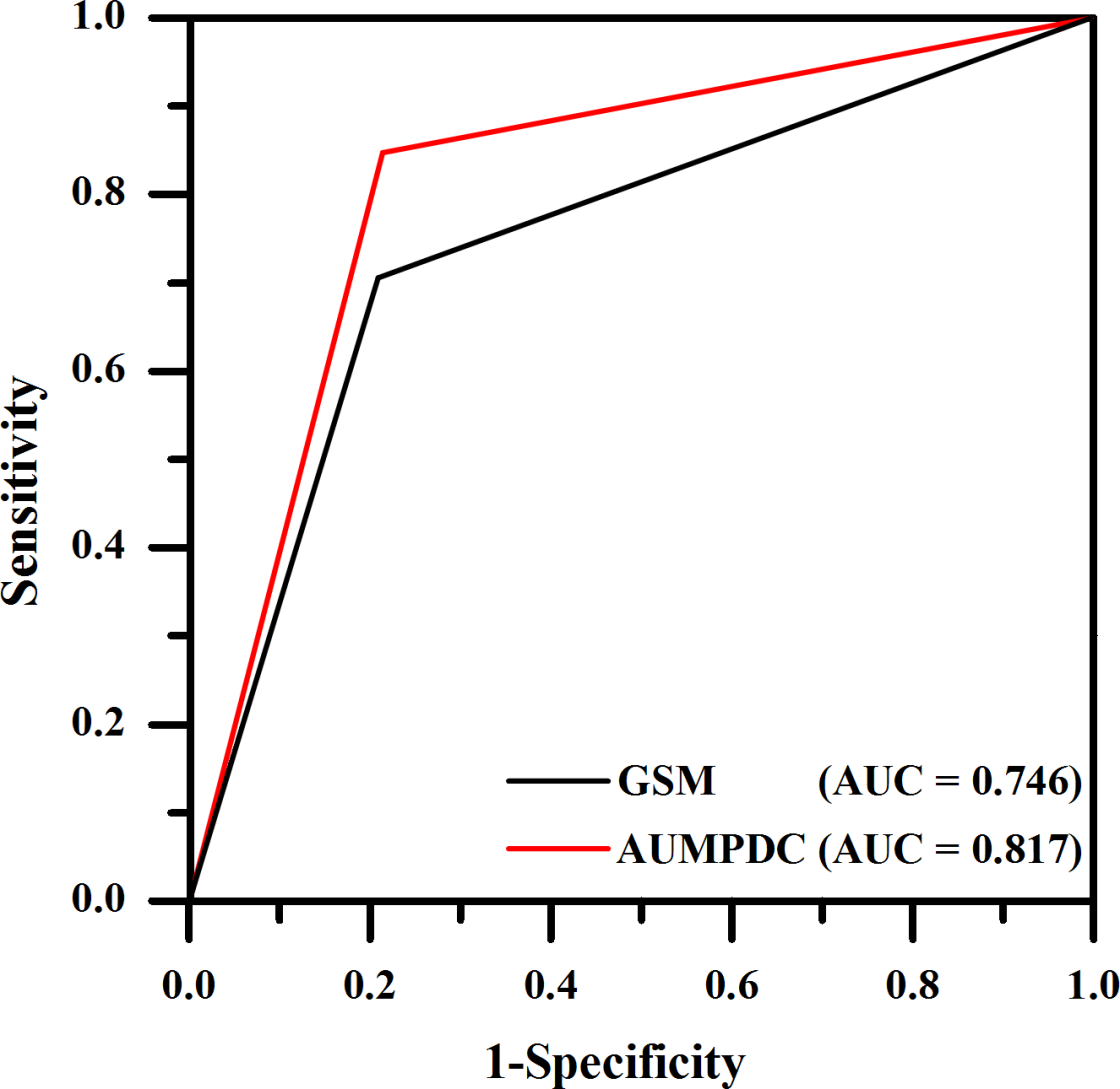
Receiver operating characteristic curves of identifying echolucent plaques for KNN classifier using AUCPDC and GSM. Note that AUC = area under the curve; GSM = gray-scale median; AUCPDC = area under cumulative probability distribution curve.

As shown in Tables 3 and 4, when AUCPDC was used for training classifier, the classification accuracy of discriminating the echo-rich, intermediate and echolucent plaques was 78.4% (*κ* = 0.673), which was higher than that obtained by using GSM 64.8% (*κ* = 0.460). When classification based on GSM, 8 of 35 intermediate plaques were classified correctly, and 21 of 35 intermediate plaques were misclassified as echolucent plaques (Table 3). The AUCPDC was more effective in discriminating intermediate and echolucent plaques than GSM. Table 4 indicated that 21 of 35 intermediate plaques were classified correctly, and 11 of 35 intermediate plaques were misclassified as echolucent plaques, when classification based on AUCPDC. Further, ROC curve analysis was developed to test the effectiveness of AUCPDC in the identification of echolucent plaques. The area under the curve (AUC) was 0.817 when AUCPDC was used for training the classifier, whereas AUC was 0.746 when GSM was used (Fig 3).

**Table 3:** Classification of 125 carotid plaques based on GSM.

		GSM			
		Echoluent	Intermediate	Echo-rich	Total
**Sonographers**	**Echoluent**	37	8	2	47
	**Intermediate**	21	8	6	35
	**Echo-rich**	2	5	36	43
	**Total**	60	21	44	125

*κ* = 0.460.

**Table 4:** Classification of 125 carotid plaques based on AUCPDC.

		AUCPDC			
		Echoluent	Intermediate	Echo-rich	Total
**Sonographers**	**Echoluent**	37	10	0	47
	**Intermediate**	11	21	3	35
	**Echo-rich**	1	2	40	43
	**Total**	49	33	43	125

*κ* = 0.673.

### E. Intra-operator agreement

**Fig 4:**
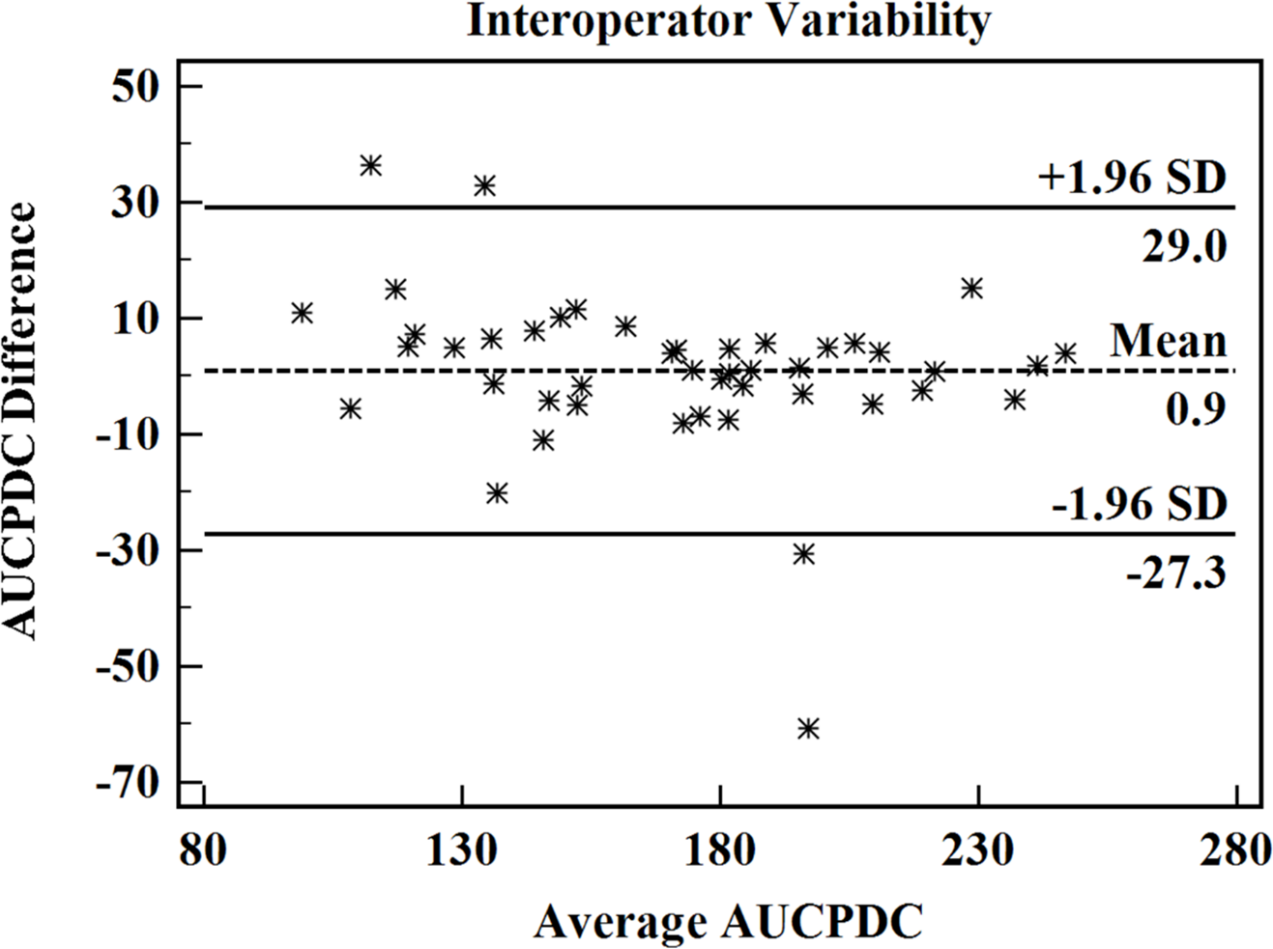
Scatterplot of the average AUCPDC against the AUCPDC difference for 45 plaques analyzed at 2 different times within a 2-month period by one operator. Note that AUCPDC = area under cumulative probability distribution curve.

Fig 4 illustrates the scatterplot of the average AUCPDC against the AUCPDC difference for 45 plaques that are analyzed at two different times within a 2-month period by one operator. Of the 45 points, there were 41 scattered in the Mean ± 1.96 SD region (95% confidence level). The AUCPDC analysis showed a good intra-operator agreement.

### F. Relationship between GSM, AUCPDC and hypertension, diabetes, CHD

Spearman's rank correlation analysis was implemented to examine the relationship between the GSM, AUCPDC and the status of hypertension, diabetes, CHD (Table 5). Compared with GSM, the AUCPDC showed a statistical association with CHD (Spearman *r* = -0.121, *p* = 0.315 vs. *r* = 0.234, *p* = 0.050).

**Table 5:** Spearman's rank correlation between the GSM, AUCPDC and the status of hypertension, diabetes, CHD.

		Hypertension	Diabetes	CHD
**GSM**	Spearman *r*	-0.031	-0.071	-0.121
	*p*	0.798	0.554	0.315
**AUCPDC**	Spearman *r*	0.1	-0.035	.234
	*p*	0.402	0.770	0.050*

GSM = gray-scale median; AUCPDC = area under cumulative probability distribution curve, CHD = coronary heart disease.

### G. Estimating Youden's index *J* in bootstrapping samples

When bootstrap was performed to estimate the Youden's index *J*, a total of 2000 bootstrapping samples was generated. The Youden’s index *J* was 0.633 (95% CI: 0.479–0.753) when AUCPDC was used to train the classifier, whereas Youden’s index *J* was 0.492 (95% CI: 0.326–0.633) when GSM was used.

## Discussion

In the present study, the AUCPDC was proposed to evaluate plaque echogenicity. Our results indicated that it is feasible to classify echo-rich, intermediate and echolucent plaques based on AUCPDC. The classification accuracy was 78.4% (*κ* = 0.673), when AUCPDC was used to train the classifier. Previous studies have proven that the echolucent plaque is a high risk indicator of cardiovascular events [8,10,18,31,32]. Compared with GSM, the AUCPDC showed a higher potential feasibility for identifying echolucent plaques (AUC = 0.817) (Fig 3), and it was more related to CHD (Spearman *r* = 0.234, *p* = 0.050) (Table 5). These indicate that AUCPDC analysis of the ultrasound images of carotid plaques might have potential in predicting the cardiovascular risk in patients with plaques.

Many studies have shown that visual classification is a feasible and reliable method for classification of ultrasound plaques with different echogenicity [12,13,33]. Geroulakos et al. classified 70 carotid plaques into five different types (type 1 uniformly echolucent, type 2 predominantly echolucent, type 3 predominantly echogenic, type 4, uniformly echogenic, and type 5 calcified plaques) and the between-observer reproducibility was 85.71% (*κ* = 0.79). Mayor et al. analyzed 95 carotid bifurcation plaques with the same five-type classification system, and a between-observer reproducibility of 91% (*κ* = 0.87) was observed. In this study, the plaques were classified into three different types (type 1 echo-rich, type 2 intermediate and type 3 echolucent) according to the scheme of the European carotid plaque study group [12], and a higher between-observer reproducibility of 96.15% (*κ* = 0.942) was achieved.

**Fig 5:**
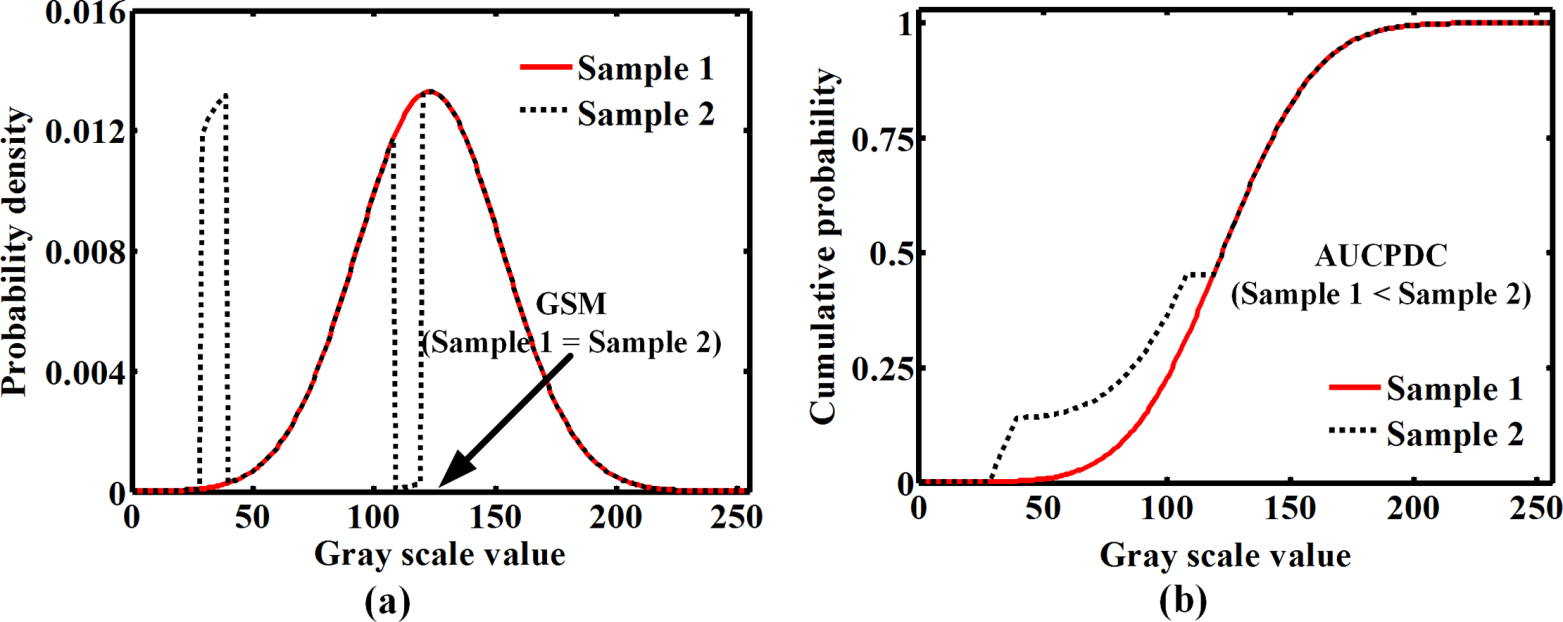
A comparison between AUCPDC with GSM when the probability density curve is changed. Note that GSM = gray-scale median; AUCPDC = area under cumulative probability distribution curve.

Both GSM and AUCPDC are calculated based on the gray scale distribution. Compared with GSM, the AUCPDC calculation takes into consideration the probability density distribution of gray scale values ranging from 0 to 255. The lower gray scale value with a high probability density gives rise to a higher value of AUCPDC. Fig 5 illustrates that the sample 1 has a same GSM with sample 2 (GSM = 128), but the AUCPDC of sample 2 is larger than sample 1. It may imply that AUCPDC is more effective than GSM in distinguishing the differences in the probability density distributions of gray scale value of plaques. Consistent with the above analysis, Tables 3 and 4 indicate that AUCPDC shows an obvious superiority in discriminating intermediate and echolucent plaques.

In this research, compared with GSM, the AUCPDC showed a statistical association with CHD (*p* = 0.05) (Table 5), but the correlation is barely significant. Such results may be caused by the relatively small sample size and sample difference. Among a total of 72 patients in this study, only 27 (37.5%) patients had CHD. What’s more, there are some methodological limitations on the calculation of AUCPDC. The ultrasound system settings (e.g. system gain, time gain compensation etc.) impact the brightness and contrast of the B-mode images, which will cause calculation bias in AUCPDC. To reduce the impact of instrument settings, normalization of the dynamic range is done by considering minimum and maximum pixel intensities within the region-of-interest. Overall, the AUCPDC is an effective parameter for evaluating the plaque echogenicity.

## Conclusion

Compared with GSM, the AUCPDC is more effective in classifying three types of plaques and identifying echolucent plaques, suggesting that AUCPDC analysis is a promising method for evaluating plaque echogenicity and predicting cardiovascular risk in patients with plaques. However, this study is limited by the relatively small number of plaques, thus future research is required to further validate our results. Furthermore, a longitudinal, prospective study utilizing carotid ultrasound examination in a large number of patients with atherosclerotic risk is required to assess the value of AUCPDC in predicting the future cardiovascular events.

## Supporting information

S1 Supporting Information Baseline characteristics. (XLSX)

S2 Supporting Information Matlab code. Classification base on a leave-one-out strategy.
(M) Click here for additional data file.

## References

[1] Yusuf S, Reddy S, Ounpuu S, Anand S (2001) Global burden of cardiovascular diseases: part I: general considerations, the epidemiologic transition, risk factors, and impact of urbanization. Circulation 104: 2746–2753. 11723030 10.1161/hc4601.099487

[2] Mozaffarian D, Benjamin EJ, Go AS, Arnett DK, Blaha MJ, Cushman M, et al (2015) Heart disease and stroke statistics—2015 update: a report from the American Heart Association. Circulation 131: e29–322. doi: 10.1161/CIR.0000000000000152 25520374 10.1161/CIR.0000000000000152

[3] Mathiesen EB, Bonaa KH, Joakimsen O (2001) Echolucent plaques are associated with high risk of ischemic cerebrovascular events in carotid stenosis: The Tromso study. Circulation 103: 2171–2175. 11331258 10.1161/01.cir.103.17.2171

[4] Ruiz-Ares G, Fuentes B, Martinez-Sanchez P, Diez-Tejedor E (2014) A prediction model for unstable carotid atheromatous plaque in acute ischemic stroke patients: proposal and internal validation. Ultrasound Med Biol 40: 1958–1965. doi: 10.1016/j.ultrasmedbio.2014.04.015 25023112 10.1016/j.ultrasmedbio.2014.04.015

[5] Nordestgaard BG, Gronholdt ML, Sillesen H (2003) Echolucent rupture-prone plaques. Curr Opin Lipidol 14: 505–512. doi: 10.1097/01.mol.0000092628.86399.9f 14501590 10.1097/01.mol.0000092628.86399.9f

[6] Gronholdt ML, Wiebe BM, Laursen H, Nielsen TG, Schroeder TV, Sillesen H (1997) Lipid-rich carotid artery plaques appear echolucent on ultrasound B-mode images and may be associated with intraplaque haemorrhage. Eur J Vasc Endovasc Surg 14: 439–445. 9467517 10.1016/s1078-5884(97)80121-9

[7] Roy J, Hedin U (2016) Commentary on 'plaque echolucency and the risk of ischaemic stroke in patients with asymptomatic carotid stenosis within the first asymptomatic carotid surgery trial (ACST-1)'. Eur J Vasc Endovasc Surg 51: 622 doi: 10.1016/j.ejvs.2016.01.002 26919935 10.1016/j.ejvs.2016.01.002

[8] Honda O, Sugiyama S, Kugiyama K, Fukushima H, Nakamura S, Koide S, et al (2004) Echolucent carotid plaques predict future coronary events in patients with coronary artery disease. J Am Coll Cardiol 43: 1177–1184. doi: 10.1016/j.jacc.2003.09.063 15063426 10.1016/j.jacc.2003.09.063

[9] Hirano M, Nakamura T, Kitta Y, Sano K, Kodama Y, Kobayashi T, et al (2010) Assessment of carotid plaque echolucency in addition to plaque size increases the predictive value of carotid ultrasound for coronary events in patients with coronary artery disease and mild carotid atherosclerosis. Atherosclerosis 211: 451–455. doi: 10.1016/j.atherosclerosis.2010.03.003 20362290 10.1016/j.atherosclerosis.2010.03.003

[10] Nakamura T, Kitta Y, Uematsu M, Sugamata W, Hirano M, Fujioka D, et al (2013) Ultrasound assessment of brachial endothelial vasomotor function in addition to carotid plaque echolucency for predicting cardiovascular events in patients with coronary artery disease. Int J Cardiol 167: 555–560. doi: 10.1016/j.ijcard.2012.01.064 22326513 10.1016/j.ijcard.2012.01.064

[11] Doonan RJ, Dawson AJ, Kyriacou E, Nicolaides AN, Corriveau MM, Steinmetz OK, et al (2013) Association of ultrasonic texture and echodensity features between sides in patients with bilateral carotid atherosclerosis. Eur J Vasc Endovasc Surg 46: 299–305. doi: 10.1016/j.ejvs.2013.05.024 23849798 10.1016/j.ejvs.2013.05.024

[12] European Carotid Plaque Study Group (2011) Reprinted article "Carotid artery plaque composition—relationship to clinical presentation and ultrasound B-mode imaging". Eur J Vasc Endovasc Surg 42 Suppl 1: S32–S38. 21855017 10.1016/j.ejvs.2011.06.022

[13] Mayor I, Momjian S, Lalive P, Sztajzel R (2003) Carotid plaque: comparison between visual and grey-scale median analysis. Ultrasound Med Biol 29: 961–966. 12878241 10.1016/s0301-5629(03)00905-0

[14] Prahl U, Holdfeldt P, Bergstrom G, Fagerberg B, Hulthe J, Gustavsson T (2010) Percentage white: a new feature for ultrasound classification of plaque echogenicity in carotid artery atherosclerosis. Ultrasound Med Biol 36: 218–226. doi: 10.1016/j.ultrasmedbio.2009.10.002 20018430 10.1016/j.ultrasmedbio.2009.10.002

[15] Christodoulou CI, Pattichis CS, Pantziaris M, Nicolaides A (2003) Texture-based classification of atherosclerotic carotid plaques. IEEE Trans Med Imaging 22: 902–912. doi: 10.1109/TMI.2003.815066 12906244 10.1109/TMI.2003.815066

[16] Tsiaparas NN, Golemati S, Andreadis I, Stoitsis JS, Valavanis I, Nikita KS (2011) Comparison of multiresolution features for texture classification of carotid atherosclerosis from B-mode ultrasound. IEEE Trans Inf Technol Biomed 15: 130–137. doi: 10.1109/TITB.2010.2091511 21075733 10.1109/TITB.2010.2091511

[17] Acharya UR, Sree SV, Krishnan MM, Molinari F, Saba L, Ho SY, et al (2012) Atherosclerotic risk stratification strategy for carotid arteries using texture-based features. Ultrasound Med Biol 38: 899–915. doi: 10.1016/j.ultrasmedbio.2012.01.015 22502883 10.1016/j.ultrasmedbio.2012.01.015

[18] Irie Y, Katakami N, Kaneto H, Takahara M, Nishio M, Kasami R, et al (2013) The utility of ultrasonic tissue characterization of carotid plaque in the prediction of cardiovascular events in diabetic patients. Atherosclerosis 230: 399–405. doi: 10.1016/j.atherosclerosis.2013.08.015 24075774 10.1016/j.atherosclerosis.2013.08.015

[19] Salem MK, Bown MJ, Sayers RD, West K, Moore D, Nicolaides A, et al (2014) Identification of patients with a histologically unstable carotid plaque using ultrasonic plaque image analysis. Eur J Vasc Endovasc Surg 48: 118–125. doi: 10.1016/j.ejvs.2014.05.015 24947079 10.1016/j.ejvs.2014.05.015

[20] Huang X, Zhang Y, Qian M, Meng L, Xiao Y, Niu L, et al (2016) Classification of carotid plaque echogenicity by combining texture features and morphologic characteristics. J Ultrasound Med 35: 2253–2261. doi: 10.7863/ultra.15.09002 27582533 10.7863/ultra.15.09002

[21] Shankar PM, Forsberg F, Lown L (2003) Statistical modeling of atherosclerotic plaque in carotid B mode images—a feasibility study. Ultrasound Med Biol 29: 1305–1309. 14553808 10.1016/s0301-5629(03)00983-9

[22] Expert Committee on the Diagnosis and Classification of Diabetes Mellitus (2003) Report of the expert committee on the diagnosis and classification of diabetes mellitus. Diabetes Care 26 Suppl 1: S5–20. 12502614 10.2337/diacare.26.2007.s5

[23] Goldenberg R, Punthakee Z (2013) Definition, classification and diagnosis of diabetes, prediabetes and metabolic syndrome. Can J Diabetes 37 Suppl 1: S8–11. 24070969 10.1016/j.jcjd.2013.01.011

[24] Sabetai MM, Tegos TJ, Nicolaides AN, Dhanjil S, Pare GJ, Stevens JM (2000) Reproducibility of computer-quantified carotid plaque echogenicity: Can we overcome the subjectivity? Stroke 31: 2189–2196. 10978050 10.1161/01.str.31.9.2189

[25] Bland JM, Altman DG (1986) Statistical methods for assessing agreement between two methods of clinical measurement. Lancet 1: 307–310. 2868172

[26] Youden WJ (1950) Index for rating diagnostic tests. Cancer 3: 32–35. 15405679 10.1002/1097-0142(1950)3:1<32::aid-cncr2820030106>3.0.co;2-3

[27] Heider P, Pfaffle N, Pelisek J, Wildgruber M, Poppert H, Rudelius M, et al (2010) Is serum pregnancy-associated plasma protein a really a potential marker of atherosclerotic carotid plaque stability? Eur J Vasc Endovasc Surg 39: 668–675. doi: 10.1016/j.ejvs.2010.03.012 20399126 10.1016/j.ejvs.2010.03.012

[28] Giede-Jeppe A, Bobinger T, Gerner ST, Madzar D, Sembill J, Lucking H, et al (2016) Lymphocytopenia is an independent predictor of unfavorable functional outcome in spontaneous intracerebral hemorrhage. Stroke 47: 1239–1246. doi: 10.1161/STROKEAHA.116.013003 27073240 10.1161/STROKEAHA.116.013003

[29] Efron B (1992) Bootstrap methods: another look at the jackknife Breakthroughs in statistics: Springer pp. 569–593.

[30] Efron B, Tibshirani RJ (1994) An introduction to the bootstrap: CRC press.

[31] Gronholdt ML, Nordestgaard BG, Schroeder TV, Vorstrup S, Sillesen H (2001) Ultrasonic echolucent carotid plaques predict future strokes. Circulation 104: 68–73. 11435340 10.1161/hc2601.091704

[32] Reiter M, Effenberger I, Sabeti S, Mlekusch W, Schlager O, Dick P, et al (2008) Increasing carotid plaque echolucency is predictive of cardiovascular events in high-risk patients. Radiology 248: 1050–1055. doi: 10.1148/radiol.2483071817 18710994 10.1148/radiol.2483071817

[33] Geroulakos G, Ramaswami G, Nicolaides A, James K, Labropoulos N, Belcaro G, et al (1993) Characterization of symptomatic and asymptomatic carotid plaques using high-resolution real-time ultrasonography. Br J Surg 80: 1274–1277. 8242296 10.1002/bjs.1800801016

